# Nursing home care for people with dementia and residents' quality of life, quality of care and staff well-being: Design of the Living Arrangements for people with Dementia (LAD) - study

**DOI:** 10.1186/1471-2318-11-11

**Published:** 2011-03-17

**Authors:** Bernadette M Willemse, Dieneke Smit, Jacomine de Lange, Anne Margriet Pot

**Affiliations:** 1 Netherlands Institute of Mental Health and Addiction (Trimbos-Institute), Utrecht, The Netherlands; 2 Department of Clinical Psychology, VU University, Amsterdam, The Netherlands

## Abstract

**Background:**

There is limited information available on how characteristics of the organization of nursing home care and especially group living home care and staff ratio contribute to care staff well being, quality of care and residents' quality of life. Furthermore, it is unknown what the consequences of the increasingly small scale organization of care are for the amount of care staff required in 2030 when there will be much more older people with dementia.

**Methods/Design:**

This manuscript describes the design of the 'Living Arrangements for people with Dementia study' (LAD-study). The aim of this study is to include living arrangements from every part of this spectrum, ranging from large scale nursing homes to small group living homes. The LAD-study exists of quantitative and qualitative research. Primary outcomes of the quantitative study are wellbeing of care staff, quality of care and quality of life of residents. Furthermore, data concerning staff ratio and characteristics of the living arrangements such as group living home care characteristics are assessed. To get more in-depth insight into the barriers and facilitators in living arrangements for people with dementia to provide good care, focus groups and Dementia Care Mapping are carried out.

**Discussion:**

Results of this study are important for policymakers, directors and staff of living arrangements providing nursing home care to people with dementia and essential for the development of methods to improve quality of care, residents' and staff well-being. Data collection will be repeated every two years, to generate knowledge on the results of changing policies in this field.

## Background

There is a growing attention for group living home care for people with dementia around the world. In the Netherlands, at the moment 25% of the residents with dementia who receive nursing home care lives in group living homes [1]. Group living home care refers to a concept of care in which nursing home care is organized in a home-like environment where residents live together in small groups. Personal care is integrated into daily routines, so that daily life is as 'normal' as possible. This means that health care staff performs care tasks as well as domestic tasks, such as cooking and cleaning [2]. In general, it is believed that group living home care is beneficial for the physical and psychosocial wellbeing of the residents [3].

Initially, group living homes were developed according to the ideals of the pioneers in this field who stated that a group living home is located in an archetypical house where a maximum of six residents live together [2]. Nowadays, several types of living arrangements providing group living home care appear. Group living home care is not only provided in archetypical houses in regular neighborhoods, but also in homes on the site of a nursing home or assisted livings and within larger scale nursing homes. The number of residents per group is no longer limited to a maximum of six. In conclusion, characteristics of group living home care are increasingly integrated - at least to some extent - in all types of living arrangements for people with dementia.

Although it is generally believed that group living home care is better for the well-being of the residents and caregivers, worries exist that group living home care is more expensive and that more staff is needed in arrangements in which many characteristics of group living home care are integrated. These worries are based on the dejuvenation in combination with the graying of the population. The prevalence of dementia will increase in the next decades [4], while the labor force will shrink in the same period of time [5]. Around 21% (N = 50.000) of all people with dementia in the Netherlands is living in a nursing home care setting [6]. It is estimated that in 2030 there would be 35 potential employees for one person with dementia, compared to 55 at this time [7]. Probably this is even an underestimation because the impact of aging of the population on the labor force was less obvious at the time these estimations were made.

Regarding the implementation of group living home care and the dejuvenation and graying of the population, it is increasingly important to get insight in what contributes to job satisfaction and burnout, sickness leave and turnover of care staff in living arrangements for people with dementia. In addition, insight in the consequences of group living home care in terms of the number of staff needed is required.

The focus of research in this field has mainly been on a comparison of quality of life of residents and staff wellbeing in two or three types of arrangements [8-17]. Two studies focusing on the effect of small scale facilities in the Netherlands compared to large scale nursing homes show modest positive results of small scale facilities on some aspects of quality of life of residents with dementia, but no differences are found for overall quality of life [10,18]. Furthermore, these previous studies show that staff working in group living homes experience more job satisfaction, a higher motivation and less burnout than staff working in regular nursing homes [9,18]. There is limited knowledge on the effect of the amount of group living home care characteristics provided in all - larger or smaller - types of nursing homes, staff ratio and other characteristics of living arrangements on quality of life of residents, quality of care and staff well being.

Two studies in the United States do focus on the relationship between characteristics of the organization of care in long-term care facilities and outcomes for residents and staff [19,20]. The Collaborative Studies of Long-Term Care shows that depression is more common for participants in for-profit nursing homes than for those in other homes (nonprofit homes and residential care/assisted living) [21]. The Maryland Long-Term Care Project shows a beneficial impact of residents' privacy and a negative impact of staff turnover on resident infection and hospitalization for infection [19]. These results support the hypothesis that characteristics of the organization of care - such as care concept or philosophy of care and staff ratio - are important for outcomes related to residents and staff in nursing home care.

### Aim and main research questions

In order to get insight into the relationship between the characteristics of living arrangements for people with dementia and residents' quality of life, quality of care and staff well-being, we designed the 'Living Arrangements for people with Dementia study' (LAD-study). Several characteristics of living arrangements are studied with special attention for the impact of group living home care characteristics and staff ratio. This article describes the methods of the first measurement cycle of LAD-study. The three main research questions are:

1. What is the effect of group living home care characteristics and staff ratio on quality of life of residents, quality of care and wellbeing of care staff?

2. What is the effect of the variety in care supply for people with dementia and the growing portion of group living home care on the amount of care staff required 20 years from now?

3. What are barriers and facilitators in living arrangements for people with dementia to provide good care?

## Methods/Design

### Design

The LAD-study is an ongoing monitor which is intended to be repeated every two years. Here we describe the design of the first measurement cycle of the LAD-study which comprises a quantitative and a qualitative study. In the quantitative study staff ratio, characteristics of the organization of care, wellbeing of care staff, quality of care and quality of life of residents are measured (research question 1 & 2).

In the qualitative study, data collection via focus groups and structured observations according to the Dementia Care Mapping (DCM) method in a selection of the participating living arrangements are carried out (research question 3). The methods concerning the quantitative and qualitative study are described separately.

### Ethical considerations

In the LAD-study, the usual daily practice in nursing home care for people with dementia is studied. Health care staff is asked to complete a questionnaire concerning their situation at work and the quality of life of the residents they are taking care of. This means that data of people with dementia are collected via observation by the health care staff. For these reasons, this study does not come within the scope of the Medical Research Involving Human Subjects Act (WMO) and therefore it does not need approval. We have come to this decision after consultation of a representative of the medical-ethics committee METiGG.

Regarding the quantitative study, the health care staff that fill in self-report and observational questionnaires on residents they are taking care of are not asked for informed consent. They are free to not return the questionnaire if they do not want to participate. The questionnaires are fully anonymous.

Written consent is asked from the registered legal representatives of the residents to be observed with Dementia Care Mapping in the qualitative study. If they do not approve of the observation of their relative, the particular resident is not observed.

## Methods quantitative study

### Study population and recruitment

All 734 living arrangements that provided nursing home care for people with dementia in the Netherlands were addressed to participate in the first data collection of the LAD-study. In the Netherlands, nursing home care for people with a primary diagnosis of dementia is organized on wards which exclusively provide care to people with dementia. These wards are comparable to special care units in the United States, however, they vary to a great extent. The aim of this study was to include 150 living arrangements from every part of this spectrum. For this purpose, we distinguished five types of living arrangements: traditional large scale nursing homes, nursing home wards in a home for the aged, large nursing home where group living home care is provided, group living homes nearby the mother facility and stand alone group living homes in the community. Our aim was to select 30 living arrangements in each of these 5 categories. All living arrangements participating in this study are non-private facilities, receiving reimbursement dependent on the referral status of the resident: a regular indication or a higher indication based on a higher level of behavioral problems.

A brochure with information on the study was sent to the director of the living arrangements and a short inventory was attached. This inventory focused on some basic organizational characteristics of the arrangement, for example the amount of residents they are caring for in total and per ward. In addition, they were asked in which of the 5 types of living arrangements they would classify the living arrangement and if they were willing to participate in our study. In case there were more than 30 arrangements in a same category willing to participate, 30 arrangements were randomly selected.

In every participating living arrangement 12 residents and 15 health care staff were randomly selected to participate in the study. In this study, we focused on health care staff (i.e. nursing assistants, certified nursing assistants and registered nurses) working on a permanent basis. Temporary staff and student-nurses were excluded.

### Measures

Table 1 provides an overview of all variables that are investigated in this study: characteristics of living arrangements, residents, and care staff and outcomes of staff ratio, care staff wellbeing, quality of care and quality of life of residents.

**Table 1:** Data collection: measures and operationalization of quantitative study

Measure	Operationalization
**Living arrangement characteristics**	

Number of residents in living arrangement	Number of residents

Number of residents per living room	Number of residents

Time of existence of living arrangement	Months

Group living home care characteristics	Group living home care questionnaire [2]

Inclusion criteria at admission	Type and number of criteria

Transferring criteria	Type and number of criteria

Education for care staff	Type of education

Technological aids in care and housing	Type and number of aids

**Resident characteristics**	

Age	Years

Gender	Male or female

Length of stay	Number of months

Visits from family or friends	Frequency

Stage of dementia	FAST [26]

ADL-dependency	KATZ [28]

Neuropsychiatric symptoms	NPI-Q [41]

Referral (reimbursement)	Euro's

**Care staff characteristics**	

Age	Years

Gender	Male or female

Origin	Dutch or other

Educational level	Type of education and level (e.g. level 1 - 5)

Working hours	Hours per week

Employment in profession	Years

Length of service	Years

**Staff ratio**	

Direct caregiver	Hours per week per resident

Educational level	Hours per week per educational level per resident

Facilitating services	Fulltime equivalent

Professional services	Fulltime equivalent

Sickness leave	Percentage

**Care staff wellbeing**	

*Primary outcomes*	

Job satisfaction	Subscale job satisfaction from LQWQ [32]

Burnout complaints	UBOS [33-35]

*Secondary outcome*	

Workload	Subscale from LQWQ [32]

Autonomy	Subscale from LQWQ [32]

Social support	Subscale from LQWQ [32]

**Quality of care**	

Physical restraints	Type and number of times used per resident

Psychotropic drugs	Type and number of times used per resident

Client satisfaction	CQ-index [25]

Approach to dementia	ADQ [36]

Involvement in activities	Subscale from MDS: RAI [24]

**Quality of life**	

Quality of life	QUALIDEM [22,23]

Pain	Subscale from MDS: RAI [24]

#### Living arrangements

The following facility demographics are collected: facility type, time of existence, number of residents in the living arrangement in total and on the wards, whether inclusion criteria are taken into account at time of the admission of a new resident, provided education for care staff, sickness leave, technological aids in care and housing and group living home characteristics. Group living home characteristics are assessed using a questionnaire we developed in a previous study based on a Concept Map concerning the ideals of group living home care [2]. Furthermore, data are collected on staff ratio: the amount of direct care staff and their level of education, the ratio of other professionals such as physicians and paramedics and the amount of facilitating services such as domestic services. Finally, registrations on the use of physical restraints and psychotropic drugs are asked to get insight in the quality of care of the arrangements.

#### Residents

Quality of life and involvement in activities are the primary outcomes for the residents. Quality of life of residents is examined with the Qualidem [22,23]. This scale is especially developed for residents with dementia in nursing home facilities. It is a multi-dimensional scale which assesses nine dimensions of quality of life in dementia: Care Relationship (7 items), Positive Affect (6 items), Negative Affect (3 items), Restless Tense Behaviour (3 items), Positive Self Image (3 items), Social Relations (6 items), Social Isolation (3 items), Feeling at Home ( items) and Having Something to Do (2 items). In addition to the Qualidem, the presence of pain is observed as an indicator of quality of life using two questions of the long term care version of the Minimum Data Set (MDS) of the Resident Assessment Instrument [24].

Residents' involvement in activities is measured with the subscale Activity Pursuit of the MDS [24]. Residents' preferences on and involvement in the last three days in 20 activities are assessed using a six-point scale. Furthermore, the amount of time residents are involved in activities or are sleeping during the day is measured.

As an indicator of quality of care, client satisfaction is assessed by asking the report of the Consumer Quality Index (CQ-index) [25], a measurement of client satisfaction which every nursing home facility must assess once every two years since 2007 in the Netherlands. For people with dementia, the informal caregiver fills in this questionnaire.

The following measures are used to control for the functioning of residents. The severity or stage of dementia is measured with the Functional Assessment Staging (FAST) questionnaire [26,27], consisting of sixteen questions. The FAST Stage is the highest consecutive level of disability of the person with dementia. A higher score on the FAST procedure indicates a more advanced stage of dementia. Assistance needed with Activities of Daily Life is measured by using the Katz index of ADL [28]. The index exists of six items, a higher score on the Katz index of ADL means more dependence in ADL. Behavioral problems were measured with the abridged (paper-and-pencil) version of the Neuropsychiatric Inventory [29]. Each of the twelve items of this scale measures a psychiatric symptom. A higher score indicates greater symptomatology. Finally, demographic variables age, gender, length of stay and frequency of family visits are assessed.

#### Care staff

Job satisfaction, intention to leave and burnout complaints are the primary outcomes for care staff. Additionally, the job characteristics of the Job-Demand-Control-Support model [30,31] and the care staff approach to dementia are measured. Job satisfaction, intention to leave and the job characteristics - job demands, job control and social support [30,31] - are measured with the Leiden Quality of Work Questionnaire [32]. This questionnaire measures 11 job characteristics. The subscales measuring Job Satisfaction (3 items) and Intention to Leave (3 items) and the four subscales concerning the JDCS model are used in this study: the Work and Time Pressure (5 items) and Decision Authority subscale (4 items) respectively measuring job demands and job control and the Social Support Supervisor subscale (4 items) and the Social Support Co-workers subscale (4 items) measuring social support.

The outcome variable burnout is measured with the Dutch version of the Maslach Burnout Inventory [33], the Utrecht Burnout Scale - C [34,35]. This scale measures three components of burnout: emotional exhaustion (8 items), depersonalization (5 items) and decreased personal accomplishment (7 items). Higher scores suggest more burnout complaints.

Person-centered attitude toward people with dementia is measured using a Dutch translation of the Approach to Dementia Questionnaire [36]. This questionnaire contains nineteen attitudinal items about people with dementia. A higher score indicates a more person-centered attitude toward people with dementia.

Finally, demographic variables and variables on care staff's employment in the living arrangement are recorded.

#### Procedure

Seventeen research assistants have been extensively trained by the research team to collect the data for the first measurement cycle of the LAD-study. The facility demographics and staff ratio of the facilitating services and other professionals are provided by interviewing the manager. The interviews are audio recorded. To calculate the staff ratio of care staff, timetables of the living arrangements are used. The CQ-index is asked from the manager to get insight in client satisfaction. Additionally, registrations of the use of physical restraints and psychotropic drugs are required from the nursing home physician. Outcomes regarding members of the care staff are based on self-report questionnaires. A registered nurse (RN) or certified nursing assistant (CNA) who is most involved with a selected resident is asked to fill in the questionnaire measuring residents' characteristics, such as their quality of life. Care staff and residents are randomly selected by the research assistants.

### Statistical analysis

Descriptive statistics are used to describe characteristics of participating living arrangements, residents and care staff. Regression analyses are used to study the effects of group living home care characteristics and staff ratio on outcomes of living arrangements. For care staff and resident outcomes multilevel regression analyses are applied.

## Methods qualitative study

### Study population and recruitment

The qualitative part of this study provides more in-depth insight into facilitators and barriers for success in living arrangements for people with dementia. Using a selection of the quantitative data of the LAD-study, all living arrangements are scored on the wellbeing of the care staff, the quality of care, the residents' quality of life and the amount of health care staff. The scores on these four outcomes are transformed to percentiles and added resulting in a 'total score of success'. For every type of living arrangement, arrangements with the highest and the lowest scores are selected. The measures on which these four outcomes are based are indicated in table 2. In total ten living arrangements are included in this qualitative study.

**Table 2:** Selection criteria for qualitative study

Selection criteria	Operationalization
**Efficiency staff ratio**	

Direct caregiver	Hours per week per resident

Educational level	Hours per week per educational level per resident

Facilitating services	Fulltime equivalent

Referral (reimbursement)	Euro's

**Quality of care**	

Physical restraints	Type and number of times used per resident

Psychotropic drugs	Type and number of times used per resident

Approach to dementia	ADQ [36]

**Care staff wellbeing**	

Job satisfaction	Subscale job satisfaction from LQWQ [42]

Intention to leave	Subscale job satisfaction from LQWQ [42]

Burnout complaints	UBOS [33-35]

**Quality of life of residents**	

Quality of life	QUALIDEM [22,23]

Pain	Subscale from MDS: RAI [24]

The selected living arrangements are asked if they are willing to participate in the qualitative part of the LAD-study. If not, the living arrangement with the second highest or lowest score is selected.

### Focus groups

The first part of the qualitative study consists of focus groups from three perspectives. Focus groups are group discussions organized to explore people's views and experiences concerning a specific set of issue's (Morgan, 1997). One focus group exists of managers and healthcare professionals, one of members of the care staff and one of family members of the residents. In all three focus groups the same questions are asked focusing on what their opinion is on points of success and improvement of the living arrangements and how care staff, residents, family members, volunteers, management, finances, philosophy of care, policy and environment contribute to this. The focus groups are conducted by a conversation leader and an assistant, taped and typed up literally. The conversation leader and assistant fill in a form after every focus group. The analysis form consists of the following questions: What did this focus group contribute to answering the research question? What got your attention? What was your own role during the conversation? What are points of attention for the next time? The focus groups reports are critically read by researchers of the research team. The reports are imported in MAXQDA and coded on points of success and improvement and suggested explanations. The texts are coded by two researchers and discussed until consensus is reached. The method of constant comparison is used in which case fragments of all participating living arrangements with the same code are compared on agreement and differences.

### Dementia Care Mapping

The second part of the qualitative study consists of structured observations using the Dementia Care Mapping (DCM) method. DCM is a method developed by the Bradford Dementia Group [37] and is based on Kitwood's psychosocial theories of dementia [38]. Dementia Care Mapping is a structured method to observe people with dementia and their formal caregivers to evaluate the quality of care from the point of view of people with dementia. Data collection involves the coding of behavior and wellbeing or ill-being of the residents every five minutes, ranging from very negative (-5) to very positive (+5). Furthermore, their involvement in activities are recorded for several behavior categories. When members of the care staff are present in the room the quality of their interactions with residents are scored ranging from highly detracting to highly enhancing behavior concerning the five psychological needs as described by Kitwood [39].

In the selected living arrangements a trained mapper observes six residents in one living room during two periods of three hours. When the mapper observes in a living room where more than six residents are staying the mapper and leader make a varied selection of residents based on gender, stage of dementia and amount of disruptive behavior. Only residents that are living in the living arrangement for more than one month are observed. In addition, the mapper assesses a number of environmental features, such as environmental cues and a home-like décor. The environmental category codes are based on the DCM-Environmental Category Codes (ECCs) [40].

### Case report

For all ten cases a report on the facilitators and barriers of the living arrangements is written. These reports are send to the living arrangements and are discussed (member check). Finally, the facilitators and barriers of all cases are compared for corresponding and differing factors. Based on results of the case reports the most important facilitators and barriers for success in living arrangements for people with dementia are specified.

## Discussion

In this paper we described the design of the Living Arrangements for people with Dementia study (LAD-study), in which 136 living arrangements providing nursing home care for people with dementia participate in the first measurement cycle. The results of this study will add to the literature in a number of ways.

Instead of comparing group living homes with another type of living arrangement, we measure the extent of integration of group living home care in daily practice and staff ratio in a broad variety of facilities. Thus, we focus on the impact of group living home care characteristics and staff ratio on the satisfaction of care staff, quality of care and quality of life of residents. In addition, the combination of the quantitative and qualitative design of the LAD-study makes it possible to get in-depth information on facilitators and barriers of success in living arrangements for people with dementia. Finally, this study gives insight into the consequences of group living home care for people with dementia for the labor market of staff.

The information concerning the organization of care is important for national and local directors and staff of living arrangements for people with dementia providing nursing home care. This knowledge can be used for the development of methods to improve care for people with dementia. Finally, this information is essential for policymakers to decide which factors in nursing home care for people with dementia need special attention or need to be stimulated. In addition to this first measurement, data collection will be repeated every two years, to generate knowledge on the results of changing policies in this field.

## Competing interests

The authors declare that they have no competing interests.

## Authors' contributions

BW drafted the manuscript and helped designing the study. DS and JdL helped draft this manuscript and helped designing the study. AMP designed and supervised the study and helped to draft this manuscript. All authors read and approved the final manuscript.

## Pre-publication history

The pre-publication history for this paper can be accessed here:

http://www.biomedcentral.com/1471-2318/11/11/prepub
